# Effect of *Abelmoschus esculentus* L. (Okra) on Dyslipidemia: Systematic Review and Meta-Analysis of Clinical Studies

**DOI:** 10.3390/ijms252010922

**Published:** 2024-10-10

**Authors:** Kabelo Mokgalaboni, Wendy N. Phoswa, Tyson T. Mokgalabone, Sanele Dlamini, Ashwell R. Ndhlala, Perpetua Modjadji, Sogolo L. Lebelo

**Affiliations:** 1 Department of Life and Consumer Sciences, College of Agriculture and Environmental Sciences, University of South Africa, Florida Campus, Roodepoort 1710, South Africa; mokgak@unisa.ac.za (K.M.); ashwell.ndhlala@ul.ac.za (A.R.N.); perpetua.modjadji@mrc.ac.za (P.M.); 2 Green Biotechnologies Research Centre of Excellence, Department of Plant Production, Soil Science and Agricultural Engineering, University of Limpopo, Private Bag X1106, Sovenga 0727, South Africa; mokgalabonetebogot@gmail.com; 3 School of Chemicals and Physical Sciences, Faculty of Agriculture and Natural Science, University of Mpumalanga, Mbombela 1200, South Africa; sanzdlamz@gmail.com; 4 Non-Communicable Diseases Research Unit, South African Medical Research Council, Tygerberg, Cape Town 7505, South Africa

**Keywords:** *Abelmoschus esculentus*, okra, *Hisbiscus esculentus*, dyslipidemia, type 2 diabetes, prediabetes, hyperglycemia

## Abstract

The global prevalence of cardiovascular diseases (CVDs), including dyslipidemia and atherosclerosis, is rising. While pharmacological treatments for dyslipidemia and associated CVDs exist, not all individuals can afford them, and those who do often experience adverse side effects. Preclinical studies have indicated the potential benefits of *Abelmoschus esculentus* and its active phytochemicals in addressing dyslipidemia in rodent models of diabetes. However, there is limited clinical evidence on lipid parameters. Thus, this study aimed to assess the potential impact of *Abelmoschus esculentus* on dyslipidemia. A literature search was performed on PubMed, Scopus, and Cochrane Library for relevant trials published from inception until 11 August 2024. Data analysis was performed using Jamovi software version 2.4.8 and Review Manager (version 5.4), with effect estimates reported as standardized mean differences (SMDs) and 95% confidence intervals (CI). The evidence from eight studies with nine treatment arms showed that *Abelmoschus esculentus* reduces total cholesterol (TC), SMD = −0.53 (95% CI: −1.00 to −0.07), *p* = 0.025), compared to placebo. Additionally, triglyceride (TG) was reduced in *Abelmoschus esculentus* compared to placebo, SMD = −0.24 (95% CI: −0.46 to −0.02), *p* = 0.035. Furthermore, low-density lipoprotein (LDL) was also reduced, SMD = −0.35 (95% CI: −0.59 to −0.11), *p* = 0.004 in *Abelmoschus esculentus* versus placebo. This remedy substantially increased high-density lipoprotein (HDL), SMD = 0.34 (95% CI: 0.07 to 0.61), *p* = 0.014). *Abelmoschus esculentus* substantially improved lipid profile in prediabetes, T2D, obesity, and diabetic nephropathy. While the evidence confirms the potential benefits of *Abelmoschus esculentus* in reducing dyslipidemia, it is important for future clinical studies to standardize the effective dosage for more reliable results. Therefore, future trials should focus on these markers in well-designed trials with sufficient sample sizes. Furthermore, *Abelmoschus esculentus* can be supplemented to the diet of the relevant populations to alleviate dyslipidemia.

## 1. Introduction

Type 2 diabetes (T2D) is a chronic metabolic disorder characterized by insulin resistance and increased blood glucose levels [1]. It remains a health challenge worldwide, with developing countries suffering more from its complications. For instance, an International Diabetes Federation (IDF) report indicates that the global prevalence of T2D was about 10.5% in 2021 and is anticipated to reach 12.2% in 2045 [2]. Moreover, the IDF statistics in 2023 reported 2 million T2D-related deaths globally [1]. These rising rates suggest that the health system will be overwhelmed and worse in rural communities. Globally, the contributing factors to the rising death rate among individuals with T2D have been reported to be due to cardiovascular disease (CVD)-associated complications [3]. For instance, in T2D, the prevalence of dyslipidemia was 96.86% in Bangladesh [4], while it was reported as 94% in South Africa [5]. This condition is characterized by impaired lipid parameters, as shown by an elevated low-density lipoprotein (LDL), total cholesterol, triglycerides (TGs) and decreased high-density lipoprotein (HDL). These promote the development of atherosclerosis, further increasing the risk of cardiovascular events [6,7,8]. Insulin resistance as a precursor for T2D increases TG levels and reduces HDL, thus promoting a proatherogenic state [9,10].

Similarly, the accumulation of small and dense LDL particles also promotes the proatherogenic state. These particles can easily enter the arterial wall, thereby contributing to the formation of atherosclerotic plaques [11,12]. Therefore, it is clear that an impaired lipid profile in T2D predisposes them to atherosclerosis. These complications persist even in patients using cholesterol-lowering pharmacological drugs such as statins [13]. In 2017, Daya et al. [14] also reported a higher prevalence of dyslipidemia among T2D on statins. Although the pleiotropic effect of statins is acknowledged in other studies, recent evidence indicates that healthy and prediabetic individuals on statin treatments have an increased risk of developing T2D [15,16,17]. While the exact mechanism is poorly documented, Besseling et al. [18] in 2015 suggested that this could be attributed to the activation of LDL receptors by statins, which allows for the migration of cholesterol from the bloodstream, adipose tissue, and liver to pancreatic cells, further damaging them. Therefore, it is important to control dyslipidemia in T2D patients to prevent its exacerbation and the development of dyslipidemia-associated complications in T2D. Although the antidiabetic drugs and supplements used in T2D can potentially ameliorate hyperglycemia, we acknowledge their limitations in T2D [19,20]. For example, individuals with T2D have an increased risk of developing CVDs, including coronary artery disease, heart attack, and stroke [21,22].

Therefore, the prevention and management of T2D should be the goal of the healthcare system in order to curb the rising CVDs and associated complications. Over the years, researchers have started exploring the various alternative treatments for their safety, toxicity, and potential benefits using animal models of diabetes. This approach has been supported by the World Health Organization (WHO) as an alternative way to manage and treat T2D and associated complications [23,24]. However, it emphasizes the importance of ensuring the safety and effectiveness of traditional medicine through scientific evidence [23]. One crucial example that has been widely researched is *Abelmoschus esculentus* L., known as okra. For instance, Nabila et al. 2018 [25] demonstrated, using a rodent model of diabetes, the anti-dyslipidemic effect of *Abelmoshus esculentus.* Thus, this plant has since gained special research interest, even in a clinical setting.

Indeed, the clinical evidence using this plant has proven some potential benefits; its safety profile has been confirmed in clinical trials, and it can therefore be considered in the management of dyslipidemia among patients at risk of dyslipidemia [26,27,28]. Due to its anti-hyperglycemic and antioxidant activities, as previously reported, it is also anticipated to have antihyperlipidemic properties. *Abelmoschus esculentus* is a flowering plant belonging to the Malvaceae family [29]. While native to East Africa, it is cultivated for commercial purposes in Afghanistan, Bangladesh, Brazil, Cyprus, Ethiopia, Ghana, India, Iran, Japan, Malaysia, Pakistan, Turkey, and the Southern USA [30,31]. It contains a variety of phytochemicals such as flavonoids, terpenes, phenols, sterols, and essential amino acids such as glutamine and asparagine [32,33,34,35]. These compounds contribute to its health benefits, such as potential therapeutic effects on T2D, CVDs, and digestive diseases, including liver detoxification, antibacterial and chemo-preventive activities [36,37]. Although the above benefits are acknowledged, especially in preclinical models of diabetes, there remain some concerns about the consistency of the findings reported in previous clinical trials. Considering the crucial role of phytochemicals found in *Abelmoscus esculentus*, this study aimed to evaluate its effect on lipid profile.

## 2. Methods

This study was conducted and reported according to the preferred reporting items for systematic reviews and meta-analyses (PRISMA) guideline [38]. This study was not registered with PROSPERO, but means were made to check PROSPERO and the Cochrane Library for similar studies, and none were found. This study adhered to the predefined PICOS [39] criteria (participants—patients with T2D, prediabetes, obesity, and diabetic nephropathy; intervention—*Abelmoschus esculentus* supplements; C—participants on placebo/hypoglycemic treatment or healthy control; outcomes—change in lipid profile; S—randomized controlled trials).

### 2.1. Search Strategies

The evidence analyzed in this study was harvested from Medline, Scopus, and Cochrane Library. No language restrictions were applied to the databases, and the search was restricted to search evidence published from inception until 11 August 2024 and updated on 25 September 2024. To retrieve relevant original studies, the search was based on various keywords and medical subject heading (MeSH) terms such as okra, *Abelmoschus esculentus*, ladies finger, *Hibiscus esculentus*, ladies finger, and type 2 diabetes mellitus, as well as two boolean operators OR and AND. The exact search strategy adapted from Medline, Scopus, and Cochrane Library is presented in Supplementary File S1.

### 2.2. Study Selection

Two independent researchers (KM and WNP) thoroughly evaluated and selected retrieved studies following PICO and predefined eligibility criteria. The Kappa score was used to assess the agreement between the above researchers [40]. Studies conducted in adults living with either prediabetes, T2D, diabetic nephropathy, or obesity were considered. All studies using *Abelmoschus esculentus*, okra, or *Hibiscus esculentus* were included. Only studies with a control group were included. Only studies that reported changes in lipid profiles, including TC, TG, LDL, and HDL as outcomes, were included. However, we excluded any study that focused on different interventions, without a control group, reporting outcomes other than lipid profiles, preclinical studies (in vitro and in vivo), books, reviews, letters, conference presentations, abstracts, preprints, theses, and dissertations.

### 2.3. Data Extraction

The data extraction process was independently carried out by two researchers, namely KM and WNP. A third independent researcher (SLL) was consulted in case of discrepancy. The results were documented in a Microsoft Excel 365 (version 2406) spreadsheet designed for this task. The data extracted from each study were composed of the following variables: authors’ last names and year of publication, references, study design, country, population status and size, age and gender distribution of participants, dosage and form of treatment, duration of intervention, and overall outcomes. The evidence presented in each study was scrutinized, and personal conclusions were drawn from each study. The reference manager Mendeley (version 2.122.1; Elsevier, 2024) was used to save all retrieved records and remove duplicates.

### 2.4. Risk of Bias (ROB) and Quality Assessment

The tool used to assess the risk of bias was RoB 2.0 [41]. It was used to assess the risk of bias in all RCTs with a focus on the following domains: risk of bias resulting from the randomization process; risk of bias due to deviations from intended interventions (intervention attribution effect); absence of outcome data; risk of bias in measuring the result; risk of bias in selecting the reported outcome. For non-randomized controlled trials, the Joanna Briggs Institute (JBI) checklist was used [42].

### 2.5. Data Analysis

All data extracted included mean, standard deviation (SD), and sample size for each study in the intervention and control groups. Heterogeneity was statistically evaluated using the *I*^2^ tests [43]. An *I*^2^ <50% was regarded as low, and those >50% were regarded as substantial heterogeneity. Varsastats, the online calculator, was used to estimate mean and SD when median and range were given (http://vassarstats.net/median_range.html (accessed on 1 August 2024)). The effect of *Abelmoschus esculentus* and placebo on lipid profiles was evaluated by exploring changes in these markers at baseline to post-treatment in both treatment groups. Mean differences were calculated by subtracting the post-treatment mean from that at baseline. The change in SD was calculated using the following formula: SD change = square root (baseline SD)^2^ + (post-SD)^2^ − 2 (coefficient of correlation (r) × baseline SD × post-SD).

An r value of 0.5 was used to calculate the change in SD. Random or fixed-effect models were employed to conduct the meta-analysis based on the level of heterogeneity between studies to determine the pooled effect. The standardized mean difference (SMD) in all lipid profiles was used to report the effect estimates with 95% confidence intervals. Forest plots were used to show the findings of the meta-analysis graphically. Publication bias was evaluated through visual inspection of funnel plots, Eggers regression, and Beggs test. An Egger regression *p*-value greater than 0.05 was used to rule out the suspicions of bias. A sensitivity analysis was conducted by excluding one study at a time to verify the reliability of the effect size [44]. Meta-regression was conducted to find an association between different confounding factors with different lipid parameters. A *p*-value of 0.05 was considered statistically significant. The inter-rater reliability was assessed using Cohen’s Kappa; a score of less than 0 was regarded as poor, 0.2 as slight, 0.40 as fair, 0.60 as moderate, 0.80 as substantial, and 1.00 as perfect agreement between researchers. Jamovi software, version 2.4.8, and Review Manager, version 5.4, were used for all analyses.

## 3. Results and Discussion

### 3.1. Selection of Evidence from the Literature

**Figure 1:**
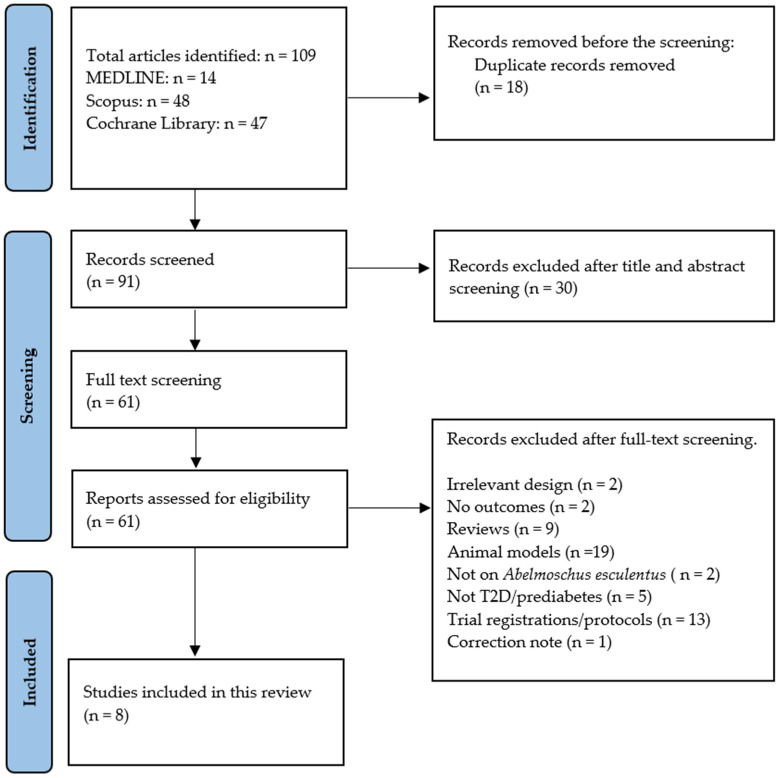
PRISMA flow chart showing the study selection process.

One hundred and nine records were sourced from Scopus, MEDLINE, and Cochrane Library (Supplementary File S2, Table S1). The kappa score for selection between KM and WNP was 0.84, with a 91.7% agreement before arbitration by the third independent researcher. However, only eight studies with nine different treatment arms were deemed relevant to address the potential impact of *Abelmoschus esculentus* in prediabetes and T2D. The selection process conducted by KM and WNP was as follows: of the 109 records identified, 18 were duplicates, and 30 had an irrelevant title, abstract, and keywords, and they were consequently excluded. Out of the remaining 61 records screened, 53 were excluded based on the following reasons: irrelevant study designs, animal models, not on prediabetes or T2D, not using *Abelmoschus esculentus*, reviews, no outcomes, trial registration/protocols, and correction notes. The study selection process is presented in Figure 1.

### 3.2. Risk of Bias (ROB) of the Included Trials

The quality of at least four studies was judged as low-risk [28,45,46,47,48,49]. One trial was classified as high-risk due to concerns related to the randomization process and selection of reported results, as no information was provided about trial registration [50] (Supplementary File S2, Figure S1). The quality of the quasi-experimental study [51] is presented in Supplementary File S2, Table S3, and the study was classified as good-quality, scoring at least 13 points out of the 14 assessed domains.

### 3.3. General Characteristics of Included Evidence

Studies [28,45,46,47,48,49,50,51] in this review were obtained from peer-reviewed journals between 2018 and 2024. The overall number of participants was 552, with at least 271 patients with T2D, 130 with impaired glucose tolerance (IGT)/prediabetes, 55 with diabetic nephropathy, and 96 overweight to moderately obese. The diagnosis of IGT was identified based on the WHO standards, with FPG levels below 7 mmol/L. Of the 552 patients, 299 patients were assigned to *Abeslmoschus esculentus*, and 253 were on placebo. The age of the recruited patients was 18 years and above. Amongst the patients treated with *Abelmoschus esculentus*, 117 were males, and 136 were females. A total of 94 male and 159 female participants were assigned to placebo. *Abelmoschus esculentus* was administered in various forms, including fruit powder, capsule, tea, or water immersion. The interventional dose ranged from 125 mg to 20000 mg (20 g). The studies were conducted in Asian regions, with four trials originating from Iran [28,45,46,47,49], one from China [50], and one from Indonesia [51], while one study was conducted in Germany [48]. The overall characteristics of the included trials are presented in Table 1.

### 3.4. Effect of Abelmoschus esculentus on Total Cholesterol (TC) Levels

**Figure 2:**
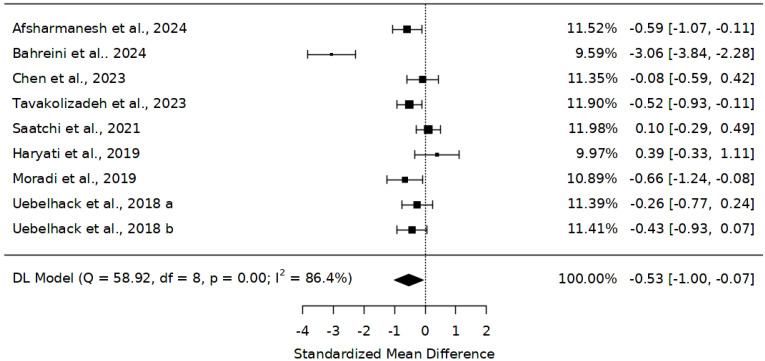
Effect of *Abelmoschus esculentus* on the level of total cholesterol [28,45,46,47,48,49,50,51]. The results are presented as standardized mean differences (SMD) and 95% confidence intervals. The black squares demonstrate the sample size in individual studies; the error bars show the confidence interval (lower and upper quartiles), and the diamond shape shows the overall effect size, a: low-dose, b: high-dose.

The potential benefits of *Abelmoschus esculentus* on TC were evaluated in seven studies with eight trials [28,45,46,47,48,49,50,51] with 587 participants (299 on *Abelmoschus esculentus* and 288 on placebo). The results of the meta-analysis exploring *Abelmoschus esculentus* showed a reduction in TC compared to placebo, SMD = −0.53 (95% CI: −1.00 to −0.07) (Figure 2). Interestingly, the average results were statistically significant (Z = −2.24, *p* = 0.025. However, the results demonstrated some heterogeneity (*I^2^* = 86.4%); thus, a random-effects model was used. The sensitivity analysis by removing one trial [49], SMD =−0.28, 95% CI (−0.51 to −0.04), Z = 0.12, *p* = 0.02.

### 3.5. Effect of Abelmoschus esculentus on Triglyceride (TG) Levels

**Figure 3:**
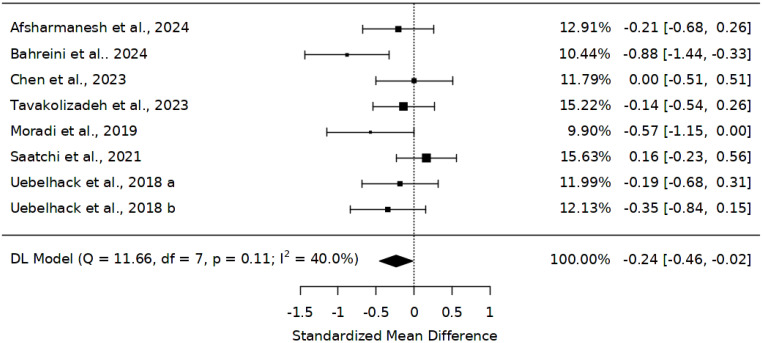
Effect of *Abelmoschus esculentus* the level of triglycerides [28,45,46,47,48,49,50]. The results are presented as standardized mean differences (SMD) and 95% confidence intervals. The black squares demonstrate the sample size in individual studies; the error bars show the confidence interval (lower and upper quartiles), and the diamond shape shows the overall effect size, a: low-dose, b: high-dose.

Evidence gathered from eight studies [28,45,46,47,48,49,50] that assessed the effect of *Abelmoschus esculentus* on triglycerides was included in this study. The overall sample size across these trials was 552, comprising 284 participants on *Abelmoschus esculentus* and 268 on placebo. The results of the meta-analysis showed a reduction in TG = −0.24 (95% CI: −0.46 to −0.02) (Figure 3). Interestingly, the average results were significant between *Abelmoschus esculentus* and placebo (Z = −2.11, *p* = 0.035). The studies showed heterogeneity (*I*^2^ = 40%); hence, a random-effects model was adopted. For sensitivity analysis, only the exclusion of Bahreini et al. [49] led to −0.14, 95% CI (−0.32 to 0.03), Z = −1.60, *p* = 0.000.

### 3.6. LDL Levels Following Abelmoschus esculentus Administration

**Figure 4:**
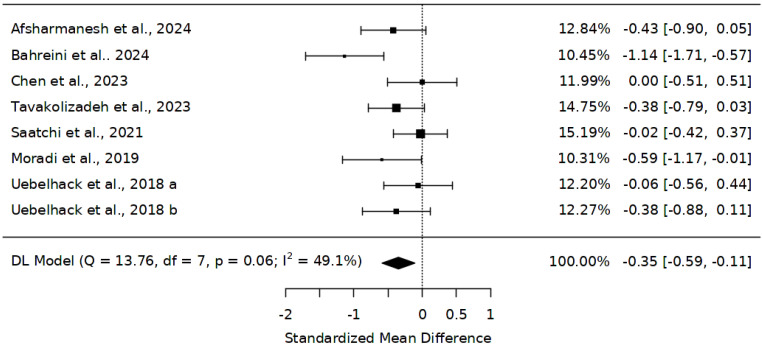
Effect of *Abelmoschus esculentus* on the level of low-density lipoprotein [28,45,46,47,48,49,50]. The results are presented as standardized mean differences (SMD) and 95% confidence intervals. The black squares demonstrate the sample size in individual studies; the error bars show the confidence interval (lower and upper quartiles), and the diamond shape shows the overall effect size, a: low-dose, b: high-dose.

Low-density lipoprotein was evaluated in eight studies [28,45,46,47,48,49,50]. The overall sample size of 552 across these trials comprised 284 participants on *Abelmoschus esculentus* and 268 on placebo. The overall effect estimates from this meta-analysis showed a reduction in LDL, SMD = −0.35 (95% CI: −0.59 to −0.11) (Figure 4). However, these findings were not significant between *Abelmoschus esculentus* and placebo (Z = −2.85, *p* = 0.004). Meanwhile, there was no evidence of heterogeneity (*I*^2^ = 49.1%). A sensitivity analysis through the exclusion of the trial by Bahreini [49] revealed a change in effect size, SMD = −0.25, 95% CI (−0.42 to −0.07), Z = −2.74, *p* = 0.001.

### 3.7. HDL Following Abelmoschus esculentus Administration

**Figure 5:**
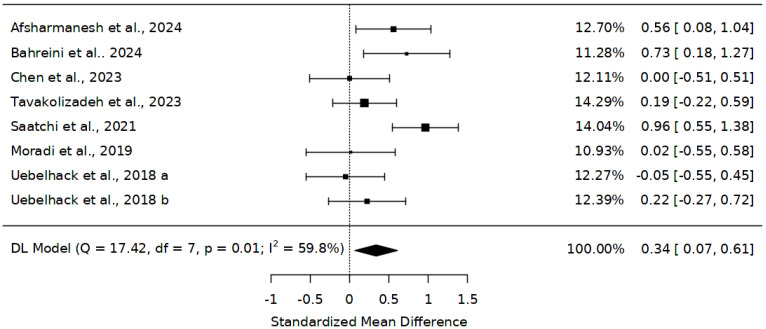
Effect of *Abelmoschus esculentus* on the level of high-density lipoprotein [28,45,46,47,48,49,50]. The results are presented as standardized mean differences (SMD) and 95% confidence intervals. The black squares demonstrate the sample size in individual studies; the error bars show the confidence interval (lower and upper quartiles), and the diamond shape shows the overall effect size, a: low-dose, b: high-dose.

High-density lipoprotein was evaluated in seven studies with eight treatment arms [28,45,46,47,48,49,50]. These studies had an overall sample size of 371, comprising 188 participants on *Abelmoschus esculentus* and 183 on placebo. The meta-analysis showed that *Abelmoschus esculentus* increased HDL compared to placebo, SMD = 0.34 (95% CI: 0.07 to 0.61) (Figure 5). However, the average results were not significant (Z = 2.46, *p* = 0.014). Meanwhile, no heterogeneity was observed, *I*^2^ = 59.8%; hence, a random-effects model was used. The exclusion of the trial by Saatchi et al. [46] led to a significant change in effect size, SMD = 0.23, 95% CI (0.03 to 0.44), Z = 2.20, *p* = 0.03.

### 3.8. Publication Bias

Publication bias in this study was visually assessed through funnel plots and statistically through Egger’s and Begg’s tests. The funnel plot regarding TC suggested no evidence of publication bias (Supplementary File S2, Figure S2A). These findings were further substantiated by the Begg and Mazumdar Rank Correlation (r = −0.167, *p* = 0.612) as presented in Supplementary File S2, Table S2. However, this was not supported by Egger’s regression test (*p* = 0.033). Similarly, for TGs, the funnel plots exhibited no indications of potential bias (Supplementary File S2, Figure S2B), a conclusion consistent with the results of the rank correlation (*p* = 0.109). However, this was not supported by the Eggers regression test (*p* = 0.008) (Supplementary File S2, Table S2). Moreover, the examination of graphical representations for both LDL (Supplementary File S2, Figure S2C) and HDL (Supplementary File S2, Figure S2D) indicated no observable publication bias, a finding supported by the statistical analyses detailed in Supplementary File S2, Table S2.

### 3.9. Meta-Regression

Mean age, sample size, patient status, country, and study design were used as moderators to explore the association with different lipid parameters. For TC only, the country where the study was conducted was revealed as a significant moderator, as it showed a significant association with a decreased TC (*p* = 0.034). No significant association was found between these moderators and the level of TG (*p* > 0.05) (Table 2). Only the study design was deemed a relevant moderator for both LDL and HDL, as demonstrated by the significant decrease and increase (Table 2).

### 3.10. Overall Discussion of the Evidence

This study analyzed clinical evidence from eight clinical studies with nine treatment arms to explore the impact of *Abelmoschus esculentus* on lipid profiles as a marker of dyslipidemia. As impaired lipid profiles predict dyslipidemia among metabolic conditions in this study, the focus was mainly on TC, TG, LDL, and HDL. The pooled evidence in this study showed that supplementation with *Abelmoschus esculentus* reduces TC, TG, and LDL and increases HDL. As increased cholesterol contributes to the development of multiple cardiovascular diseases, including atherosclerosis, the observed potential benefits of *Abelmoschus esculentus* in reducing their levels are essential in ameliorating the aforementioned complications. These results have been reported in preclinical studies. For instance, Uadia and colleagues, using a rodent model of diabetes, reported a significant reduction in TC after a diet formulated with *Abelmoschus esculentus* was given to diabetic rats compared to nondiabetic rats [52]. In addition, this study reported a significant reduction in TG, LDL, VLDL, and an increased HDL. This supports the idea that *Abelmoschus esculentus* supplementation alleviates dyslipidemia in diabetes. More recently, different extracts of *Abelmoschus esculentus* in diabetic rats resulted in a significant reduction in TC, with one exception of ethanolic extract [53]. This suggests that the *Abelmoschus esculentus* preparation method can have an impact on its overall efficacy in alleviating dyslipidemia.

Interestingly, evidence from rodent models of diabetes demonstrated a significant reduction in TC after *Abelmoschus esculentus* administration [54,55,56,57]. These results further support the view that preclinical studies could be used to test for the efficacy of herbal remedies in diabetes. On the other hand, evidence from clinical trials has also confirmed the effect of *Abelmoschus esculentus* in T2D, as a mixture of 10 g of okra powder with yogurt substantially reduced TC in T2D. Similarly, Haryati et al. [51] also showed an improved TC following 250 mL *Abelmoschus esculentus* fruit water immersion in T2D. While the latter study had a different design, a consistent effect on TC was observed. More recent evidence from RCTs still supports the potential cardioprotective properties of *Abelmoschus esculentus* in T2D. In particular, Afsharmanesh et al., 2024 [45] showed that supplementation with 500 mg of *Abelmoschus esculentus* significantly reduced TC.

While the overall evidence suggests potential benefits of *Abelmoschus esculents* in ameliorating TC in T2D and prediabetes, contrasting findings were reported by another trial that demonstrated no effect of 1000 mg of *Abelmoschus esculentus* whole-fruit capsules on TC in T2D patients [46], contradicting the preclinical and clinical results reported by previous researchers. Consistent findings were recently reported by Chen et al. [50] in 2023, who showed no effect of 20 g of dried *Hibiscus esculentus* fruit tea on TC among patients living with prediabetes. These inconsistent findings would question the effect of this plant-based remedy on dyslipidemia amongst patients living with prediabetes. These contradicting findings may be due to the differing pathogenesis of these two conditions and the difference in dosage and interventional duration of these remedies. While a beneficial effect of this supplement was observed, it is important to understand its mode of action in relation to dyslipidemia. The existing report suggests that the potential hypolipidemic properties of *Abelmoschus esculentus* are due to its direct effects on enzymes involved in cholesterol synthesis and metabolism [58]. This contributes to reduced cholesterol levels. It has also been reported that the mucus-like characteristic of *Abelmoschus esculentus* removes cholesterol by binding to it in the intestine and then excreting it from the body, thereby preventing it from being absorbed into the bloodstream [59]. It is noteworthy that T2D patients have high levels of non-esterified fatty acids (NEFAs), which are the fatty acids that circulate in the blood [60]. These levels contribute to insulin resistance, which further promotes the accumulation of TGs and lowers the clearance of LDL [61].

On the other hand, NEFAs upregulate the expression of lipogenic molecules such as sterol regulatory element-binding protein 1c (SREBP1c) and fatty acid synthase (FAS) [61]. They also decrease the expression of lipolytic molecules such as carnitine palmitoyltransferase 1A (CPT1A) and hormone-sensitive lipase (HSL) [61]. However, as noted, *Abelmoschus esculentus* has the potential to downregulate NEFAs in T2D, which in turn suppresses the synthesis of cholesterol and maximizes LDL clearance [62]. This mechanism is supported by the preclinical evidence that showed *Abelmoschus esculentus* abilities to control cholesterol metabolism by downregulating the expression of SREBP1c [58]. SREBP1c is a transcription factor that is involved in lipid metabolism. It promotes the expression of genes involved in fatty acid and TG synthesis. It also regulates the expression of enzymes and proteins involved in cholesterol synthesis and uptake [63,64].

Other researchers suggest that the hypolipidemic activity of *Abelmoschus esculentus* is associated with an upregulation of lipogenesis through the inhibition of cholesterol-7-α-hydroxylase (CYP7A1) and (FAS) [58]. CYP7A1 is an enzyme that catalyzes the conversion of cholesterol into the bile acid precursor (7α-hydroxycholesterol) [65]. On the other hand, Nabila and colleagues reported that the hypolipidemic properties of *Abelmoschus esculentus* are mediated by its high fiber content [25]. The soluble fiber in *Abelmoschus esculentus* has a high affinity to bind to bile acids and associated cholic acids [59,66]. Bile acids are the products of cholesterol in the liver and are involved in fat digestion [67]. Notably, when bile acids bind to *Abelmoschus esculentus* soluble fibers, this enhances their excretion, thus stimulating the liver to produce more bile acids from cholesterol. Therefore, the body’s excretion of the fiber–bile acid complex reduces cholesterol levels. Supporting the above findings is the evidence from hyperlipidemia-induced C57BL/6 mice-fed diets mixed with 1% or 2% *Abelmoschus esculentus* powder [58]. The study reported a decreased serum and hepatic TC and enhanced fecal excretion of bile acid. Therefore, this evidence supports the potential benefits of *Abelmoschus esculentus* in reducing TC through its effects on cholesterol metabolism, binding, and excretion. LDL and HDL are also deemed as ideal markers of dyslipidemia in T2D. While these results showed a significant effect, they are in disagreement with previous clinical studies [28,46,50] that demonstrated no effect of *Abelmoschus esculentus* on LDL and HDL. However, supporting our findings is another trial that demonstrated a decrease in LDL post-treatment, 117.28 ± 30.66 mg/dL, compared to baseline,125.44 ± 34.22 mg/dL, *p* ˂ 0.05 [47]. These results suggest a potential benefit of *Abelmoschus esculentus* in reducing LDL levels, which can be considered an important approach to ameliorating dyslipidemia. However, in another trial [46], no significant difference was observed in HDL (*p* > 0.05).

Similar findings were reported by Nabila et al., 2018 [25], who showed that Abelmoschus esculentus had no significant effect on HDL while significantly reducing LDL in a rodent model of diabetes. A comprehensive report from their qualitative study showed that the administration of *Abelmoschus esculentus* powder can significantly decrease LDL in rats with diabetes [68]. Therefore, these results, especially on HDL, suggest some potential limitations of *Abelmoschus esculentus* in regulating lipid metabolism. Interestingly, *Abelmoschus esculentus* seems to increase HDL, known as good cholesterol, by upregulating the expression of LDL receptors (LDLR), which promotes the clearance of LDL [59]. Additionally, the high polyphenols and flavonoid content in *Abelmoschus esculentus* contributes to a high level of HDL by inhibiting the oxidation of LDL [69,70]. Indeed, the findings observed in this study suggest that Abelmoschus esculentus can be used as an alternative herbal treatment to manage and control dyslipidemia.

#### Strengths and Limitation

In this study, independent, experienced researchers used major databases to gather evidence to avoid searching bias. The quality of the eight trials included was good, and only one was of fair quality. Despite the observed heterogeneity in different lipid parameters, a meta-regression was performed to assess the association between confounders such as age, sample size, patient status, country, and study design. The evidence synthesized in this study was collected from only eight studies with a small sample size (552) with a range of 15 to 99. This might limit the statistical power. The protocol for this study was not registered with PROSPERO. However, the library was searched for any similar protocols, and nothing was obtained from the databases. The evidence suggests potential benefits on all lipid parameters. The different doses of *Abelmoschus esculentus* and durations of administration were noted, and this could affect the overall interpretation of our findings. Despite the potential benefits of *Abelmoschus esculentus,* the included trials were conducted in Iran, Indonesia, China, and Germany. This could also limit the interpretation and translatability of the current findings in other countries, including African countries, where the prevalence of T2D and metabolic disorders is high. The availability of *Abelmoschus esculentus* in Asian countries might have contributed to this skewness of data compared to other countries. Quality control of the plant was not reported in any of the trials, which could also limit our interpretation.

## 4. Conclusions

In conclusion, the clinical evidence in this study suggests that treating T2D and prediabetes, obesity, and diabetic nephropathy with *Abelmoschus esculentus* might offer potential benefits by ameliorating dyslipidemia. Based on these findings, we recommend that these groups of patients use *Abelmoschus esculentus* to supplement their diet to ameliorate dyslipidemia. However, this should be closely monitored, especially in those who are using other antilipidemic and hyperglycemic drugs, to avoid drug-to-herb interaction that can render treatment less effective. Therefore, based on these results, we recommend that future trials with good methodological qualities and sufficient sample sizes be conducted, and these should focus on assessing the effect of *Abelmoschus esculentus* on lipid parameters and the associated lipid regulator genes, as well as proteins such as apolipoprotein-E (APOE), cholesteryl ester transfer protein (CETP), lipoprotein lipase (LPL), and LDLR. These could assist in ascertaining the overall effect of *Abelmoschus esculentus* on lipid metabolism and dyslipidemia. The form, dose, and intervention period should be standardized for optimal benefits among these populations. The lack of clinical trials exploring *Abelmoschus esculentus* in African countries warrants future trials to focus on this treatment in this population. This will help to curb the rising burden of CVD amongst these populations.

## Figures and Tables

**Table 1:** Basic characteristics of included participants across all studies.

Leading Author and Year	Study Design	Country	Sample Size and Treatment Groups of Participants	Abelmoschus Esculentus Group BMI (kg/m^2^)	Age of Participants (years)	Gender (m/f) Treatment/Placebo	Intervention and Period	Summary of Findings
Tavakolizadeh et al. 2023 [28]	Double-blind, randomized, parallel, placebo-controlled trial	Iran	94 patients with T2D 48 on okra 46 on placebo	28.6 ± 2.05	40–65	17/31 13/33	Six capsules containing 500 mg of powdered okra fruit three times a day for three months	Significant reduction in TC and TG without changes in LDL and HDL.
Afsharmanesh et al. 2024 [45]	Double-blind, randomized, placebo-controlled clinical trial	Iran	70 patients with prediabetes 35 on okra 35 on placebo	NR	30–35	15/20 15/20	Two capsules containing 500 mg of okra three times a day for eight weeks	Significant decrease in TC and LDL and increased HDL without changes in TG.
Bahreini et al. 2024 [49]	Triple-blind, randomized, placebo-controlled clinical trial	Iran	55 patients with diabetic nephropathy 30 on okra 25 on placebo	NR	40–70	20/10 19/6	125 mg of dried okra extract for ten weeks	Significant decrease in TG and increase in HDL post okra treatment.
Saatchi et al. 2021 [46]	Double-blind, randomized clinical trial	Iran	99 patients with T2D 50 on *Abelmoschus esculentus* 49 on placebo	30.2 ± 4.3	18 and above	23/26 13/37	1000 mg of *Abelmoschus esculentus* whole-fruit capsules orally every 6 h for eight weeks	No significant changes in TC, TG, LDL, and HDL.
Moradi et al. 2019 [47]	Double-blind, single-center, randomized clinical trial	Iran	48 patients with T2D 25 on okra 23 on placebo	24.90 ± 3.94	30–75	9/21 7/23	10 g okra powder blended in 150 g conventional yogurt for eight weeks	Significantly decreased TG, TC, and LDL without significant change in HDL.
Chen et al. 2023 [50]	RCT	China	60 patients with IGT 30 on *Hibiscus esculentus* 30 on placebo	NR	Above 75	18/12 14/16	20 g of *Hibiscus esculentus* dried fruit tea daily for 60 days	No significant changes in TC, TG, LDL, and HDL.
Uebelhack et al. 2018 a [48]	Double-blind, randomized, placebo-controlled trial	Germany	62 overweight to moderately obese patients 32 okra 30 placebo	29.15 ± 2.17	18–65	6/27 9/22	Two capsules of 165 mg dehydrated okra powder three times a day for 12 weeks	No significant difference in lipid profile after treatment.
Uebelhack et al. 2018 b [48]	Double-blind, randomized, placebo-controlled trial	Germany	64 overweight to moderately obese patients 34 on okra 30 on placebo	29.44 ± 2.31	18–65	9/26 9/22	Two capsules of 330 mg dehydrated okra powder three times a day for 12 weeks	No significant difference in lipid profile after treatment.
Haryati et al. 2019 [51]	Quasi-experimental	Indonesia	30 patients with T2D 15 on okra 15 on placebo	NR	31–60	4/11 4/11	Okra fruit water immersion (250 mL) once a day in the morning for two weeks	Significantly decreased TC.

RCT: randomized controlled trial; BMI: body mass index; IGT: impaired glucose tolerance; T2D: type 2 diabetes mellitus; LDL: low-density lipoprotein; HDL: high-density lipoprotein; TC: total cholesterol; TG: triglycerides; NR: not reported; a: low-dose; b: high-dose; m: male; f: female

**Table 2:** Association between total cholesterol and different moderators.

Parameters	Moderators	Effect Size	SE	95% CI	*p*-Value
Total cholesterol [28,45,46,47,48,49,50,51]	Mean age	−0.2107	0.3368	−0.8708 to 0.4493	0.5315
	Sample size	0.2297	0.4850	−0.7210 to 1.1803	0.6359
	Patient status	−1.0071	0.6819	−2.3436 to 0.3294	0.1397
	Country	−1.0038	0.4720	−1.9289 to −0.0786	0.0335 *
	Design	−0.7448	0.4649	−1.6561 to 0.1664	0.1092
Triglyceride [28,45,46,47,48,49,50]	Mean age	−0.1861	0.1726	−0.5244 to 0.1521	0.2808
	Sample size	−0.5745	0.3535	−1.2673 to 0.1183	0.1041
	Patient status	−0.2332	0.3138	−0.8483 to 0.3819	0.4574
	Country	−0.0000	0.3678	−0.7209 to 0.7209	1.0000
	Design	−0.1658	0.0976	−0.3571 to 0.0255	0.0894
LDL [28,45,46,47,48,49,50]	Mean age	−0.2202	0.1801	−0.5732 to 0.1327	0.2213
	Sample size	−0.5916	0.3913	−1.3585 to 0.1753	0.1305
	Patient status	−0.4599	0.3388	−1.1239 to 0.2041	0.1746
	Country	0.0000	0.3727	−0.7306 to 0.7306	1.0000
	Design	−0.2819	0.0964	−0.4709 to −0.0929	0.0035 *
HDL [28,45,46,47,48,49,50]	Mean age	0.1868	0.191	−0.1890 to 0.5626	0.3299
	Sample size	0.0165	0.4235	−0.8135 to 0.8465	0.9689
	Patient status	0.5106	0.3775	−0.2292 to 1.2504	0.1762
	Country	0.0000	0.3736	−0.7322 to 0.7322	1.0000
	Design	0.3335	0.1629	0.0142 to 0.6527	0.0406 *

SE: standard error, LDL: low-density lipoprotein, HDL: high-density lipoprotein. * indicates statistically significant results.

## Data Availability

All data supporting this manuscript are provided in the Supplementary Materials.

## Supplementary Materials

The following supporting information can be downloaded at https://www.mdpi.com/article/10.3390/ijms252010922/s1.
