# Supplementary material for: Effect of Abelmoschus esculentus L. (Okra) on Dyslipidemia: Systematic Review and Meta-Analysis of Clinical Studies
Source: Int J Mol Sci. 2024 Oct 10;25(20):10922. doi: 10.3390/ijms252010922 (PMC11507881; doi:10.3390/ijms252010922)
Supplement: Supplementary file 1 [file ijms-25-10922-s001.zip › ijms-3221482-supplementary file S2.pdf]

Table S1. Exact search strategies adapted on databases.

Database	Exact Search	Records
MEDLINE	((((*Abelmoschus esculentus*[MeSH Terms]) OR (Okra[MeSH Terms])) OR (*Hibiscus esculentus*[MeSH Terms])) OR (ladies finger[MeSH Terms])) AND (type 2 diabetes mellitus[MeSH Terms])	14
Cochrane Library	OKRA OR *Abelmoschus esculentus* OR *Hibiscus esculentus* OR ladies finger AND type 2 diabetes mellitus	47
Scopus	(TITLE-ABS-KEY (okra) OR TITLE-ABS-KEY (abelmoschus AND esculentus) OR TITLE-ABS-KEY (hibiscus AND esculentus) OR TITLE-ABS-KEY (ladies AND finger) AND TITLE-ABS-KEY (type 2 diabetes AND mellitus) OR TITLE-ABS-KEY (type 2 diabetes))	48

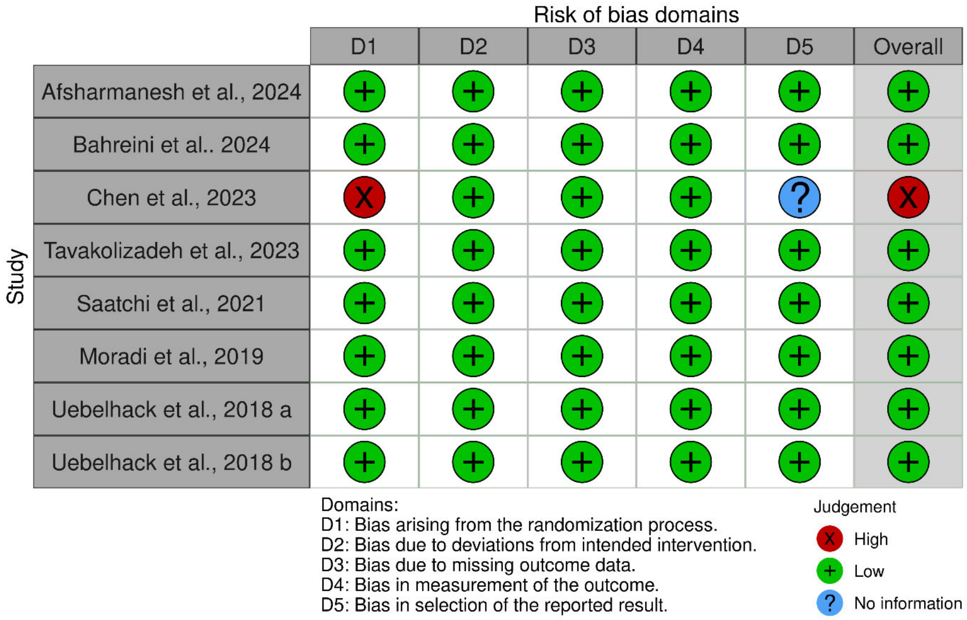

Figure S1. Risk of Bias assessment results.

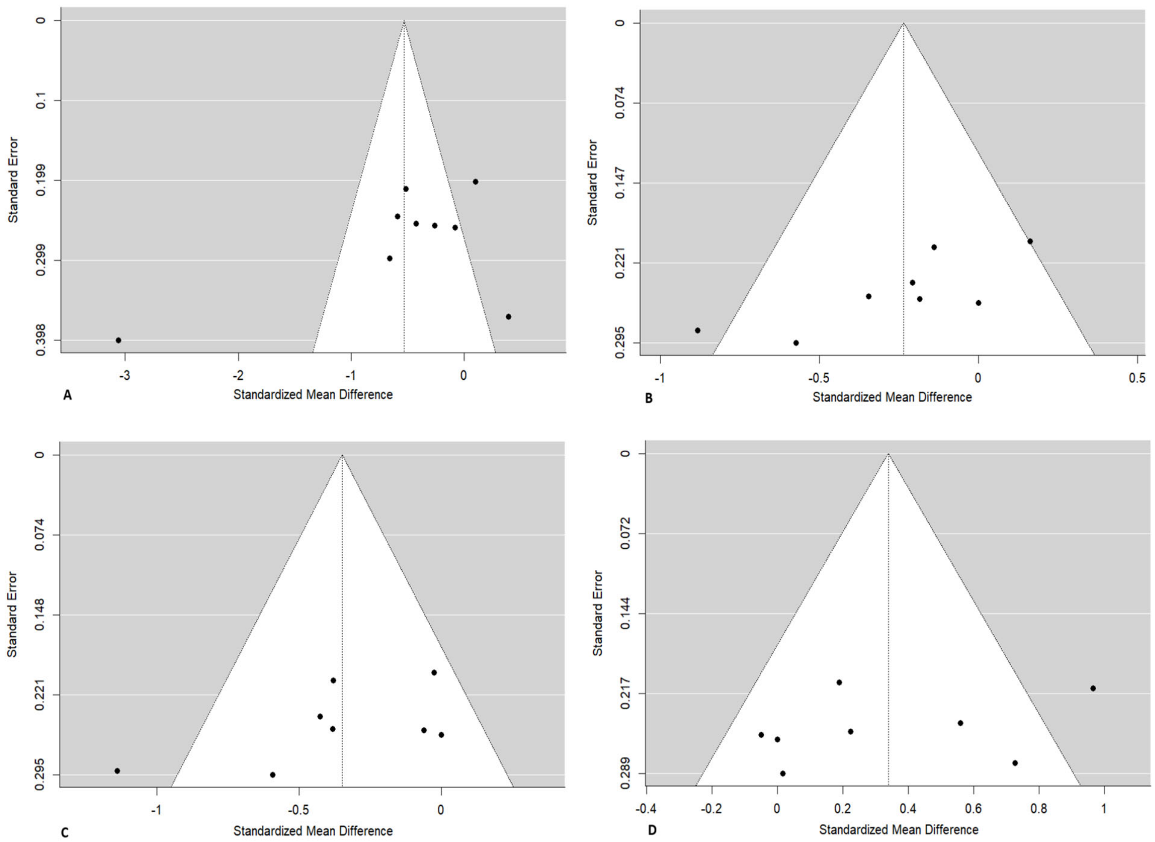

Figure S2. Publication bias across all included studies on different lipids parameters. A total cholesterol, B: triglycerides, C: low-density lipoprotein and D: high-density lipoprotein.

Table S2: Publication Bias Assessment

Test name	Lipid profiles	Value	P
Fail-Safe N	Total cholesterol	90000	<0.001
Begg and Mazumdar Rank Correlation		−0.167	0.612
Egger's Regression		−2.13	0.033
Trim and Fill Number of Studies		0.000	.
Fail-Safe N	Triglyceride	16000	0.003
Begg and Mazumdar Rank Correlation		−0.500	0.109
Egger's Regression		−2.666	0.008
Trim and Fill Number of Studies		0.000	.
Fail-Safe N	Low-density lipoprotein	40000	<0.001
Begg and Mazumdar Rank Correlation		−0.286	0.399
Egger's Regression		−1.756	0.079
Trim and Fill Number of Studies		1.000	.
Fail-Safe N	High-density lipoprotein	38000	<0.001
Begg and Mazumdar Rank Correlation		−0.214	0.548
Egger's Regression		−0.836	0.403
Trim and Fill Number of Studies		0.000	.

Table S3. Risk of bias assessment based on Joanna Briggs Institute (JBI) for the quasi-experimental study

RoB Assessor: K Mokgalaboni	Date of Appraisal: 25 September 2024	Record Number: 1
**Study Author: Haryati**	**Study Title:** The effectiveness of okra fruit (Abelmoschus esculentus) on fasting blood sugar and total cholesterol level in type 2 diabetes mellitus	**Study Year: 2019**

	Internal Validity	Choice - Comments/Justification	Yes	No	Unclear	N/A
	**Bias related to temporal precedence**					
1	Is it clear in the study what is the “cause” and what is the “effect” (i.e. there is no confusion about which variable comes first)?		☒	☐	☐	☐
	**Bias related to selection and allocation**					
2	Was there a control group?		☒	☐	☐	☐
	**Bias related to confounding factors**					
3	Were participants included in any comparisons similar?	Equal number of males (4) nd females (11) in both groups	☒	☐	☐	☐
	**Bias related to administration of intervention/exposure**					
4	Were the participants included in any comparisons receiving similar treatment/care, other than the exposure or intervention of interest?		☐	☒	☐	☐

Bias related to assessment, detection and measurement of the outcome

			Yes	No	Unclear	N/A
5	**Were there multiple measurements of the outcome, both pre and post the intervention/exposure?**					
	**Outcome 1**	Blood glucose pre and post in both groups	☒	☐	☐	☐
	**Outcome 2**	Total cholesterol pre and post in both groups	☒	☐	☐	☐

			Yes	No	Unclear	N/A
6	**Were the outcomes of participants included in any comparisons measured in the same way?**					
	**Outcome 1**		☒	☐	☐	☐
	**Outcome 2**		☒	☐	☐	☐

			Yes	No	Unclear	N/A
7	**Were outcomes measured in a reliable way?**					
	**Outcome 1**		☒	☐	☐	☐
	**Outcome 2**		☒	☐	☐	☐

Bias related to participant retention

**Was follow-up complete and if not, were differences between g in terms of their follow-up adequately described and analyzed?**						
**Outcome 1**			**Yes**	**No**	**Uncle**	**N/A**
Result 1			☒	☐	☐	☐
**Outcome 2**			**Yes**	**No**	**Uncle**	**N/A**
Result 1			☒	☐	☐	☐

Statistical Conclusion Validity

**Was appropriate statistical analysis used?**						
**Outcome 1**			**Yes**	**No**	**Uncle**	**N/A**
Result 1			☒	☐	☐	☐
**Outcome 2**			**Yes**	**No**	**Uncle**	**N/A**
Result 1			☒	☐	☐	☐

Overall appraisal: **Include:** ☒ **Exclude:** ☐ **Seek Further Info:** ☐

Comments: The study scored 13 points out of 14 hence classified as good quality
